# The relationship between cervical length and area measurements evaluated by MRI and the amount of hemorrhage in PAS cases

**DOI:** 10.1186/s12884-024-06472-5

**Published:** 2024-04-19

**Authors:** Yongfei Yue, Liping Zhu, Chengfeng Liu, Yanli Lu

**Affiliations:** 1 Department of Obstetrics and Gynecology, The Affiliated Suzhou Hospital of Nanjing Medical University, Suzhou Municipal Hospital, No. 26 Daoqian Street, Gusu District, Suzhou, Jiangsu 215002 China; 2 Department of Medical Imaging, The Affiliated Suzhou Hospital of Nanjing Medical University, Suzhou Municipal Hospital, Suzhou, Jiangsu China

**Keywords:** Complete placenta previa, Magnetic resonance imaging, Massive hemorrhage, Placenta accreta spectrum

## Abstract

**Background:**

Placenta accreta spectrum often leads to massive hemorrhage and even maternal shock and death. This study aims to identify whether cervical length and cervical area measured by magnetic resonance imaging correlate with massive hemorrhage in patients with placenta accreta spectrum.

**Methods:**

The study was conducted at our hospital, and 158 placenta previa patients with placenta accreta spectrum underwent preoperative magnetic resonance imaging examination were included. The cervical length and cervical area were measured and evaluated their ability to identify massive hemorrhage in patients with placenta accreta spectrum.

**Results:**

The cervical length and area in patients with massive hemorrhage were both significantly smaller than those in patients without massive hemorrhage. The results of multivariate analysis show that cervical length and cervical area were significantly associated with massive hemorrhage. In all patients, a negative linear was found between cervical length and amount of blood loss (r =−0.613), and between cervical area and amount of blood loss (r =−0.629). Combined with cervical length and cervical area, the sensitivity, specificity, and the area under the curve for the predictive massive hemorrhage were 88.618%, 90.209%, and 0.890, respectively.

**Conclusion:**

The cervical length and area might be used to recognize massive hemorrhage in placenta previa patients with placenta accreta spectrum.

## Introduction

The exact mechanism of placenta accreta spectrum (PAS) disorders is not fully understood, however, multiple studies have shown that previous cesarean section and placenta previa pregnancy were the most important risk factors for PAS [1–3]. Ultrasound is widely used for prenatal diagnosis and evaluation of placenta previa with PAS, but MRI has advantages that ultrasound does not, such as the ability to provide greater spatial resolution of soft tissue and clearer pelvic organ anatomic structures [4]. PAS can prevent the placenta from separating from the uterine wall during delivery, affecting the closure of the uterine sinus and uterine contractions, which may lead to life-threatening bleeding during cesarean section and postpartum, especially bleeding near the cervix. It is important to identify placenta previa with PAS patients who are at risk of massive hemorrhage (MH), which is useful for appropriate prenatal consultation and developing an individualized delivery plan. However, the severity of MH varies among patients with diagnosed PAS. To further improve the ability to identify individuals at high risk of MH, other risk factors need to be further explored.

Various MRI features have been shown satisfactory sensitivity and specificity in predicting placenta accreta in placenta previa patients before cesarean Sects. [5, 6]. The lower uterine segment and cercvix have less muscle tissue, and there is no effective contraction after placenta separation, which may lead to MH [7]. In addition, the cervix is located deep in the pelvic cavity, where the space is small, which makes it difficult to suture hemostasis [8]. Many studies have shown that cervical length had a vital impact on perinatal outcomes in patients with PAS, such as MH and peripartum hysterectomy [9, 10]. However, the correlation between cervical length, cervical area and MH in women with PAS disorders is less studied, especially the relationship between cervical area and MH. Therefore, the aim of our study is to investigate the correlation between cervical length, cervical area and MH evaluated by MRI.

## Materials and methods

### Study population and MRI protocol

This retrospective study was approved by the Ethics Review Board of the affiliated Suzhou Hospital of Nanjing Medical University (K-2022-015-K01). Informed consent was waived because of the retrospective nature of this study with anonymous selection, which did not subject the patients to new interventions. Both clinical and histologic FIGO criteria were used to classify the grade of PAS, including accreta, increta, and percreta [11]. The patients underwent prenatal MRI examination and delivered at our institution between January 2016 and December 2022. The inclusion criteria were as follows: (1) complete placenta previa pregnancies suspected with PAS disorders who evaluated by MRI, (2) patients were confirmed PAS by clinical and histologic FIGO criteria, (3) single pregnant women with a history of more than one cesarean section, (4) patients with high quality MRI image. Finally, a total of 158 patients were included in the study. In accordance with previous clinical studies [12, 13], MH is defined as maternal bleeding exceeding 2000 ml within 24 h after cesarean section.

The MRI examinations were performed on 3 T scanner (Siemens Medical Solutions, Erlangen, Germany) in a supine position without gadolinium. All patients underwent placental MRI using T_2_-weighted half-fourier acquisition single shot turbo spin echo (HASTE) sequence (repetition time 700 ms, echo time 87 ms, bandwidth 698 Hz/px, 432 × 432 matrix over a field of view of 380 × 380 mm, 5 mm slice thickness). The pregnant women were imaged in sagittal, coronal, and axial orientations with a partially full bladder.

**Fig. 1:**
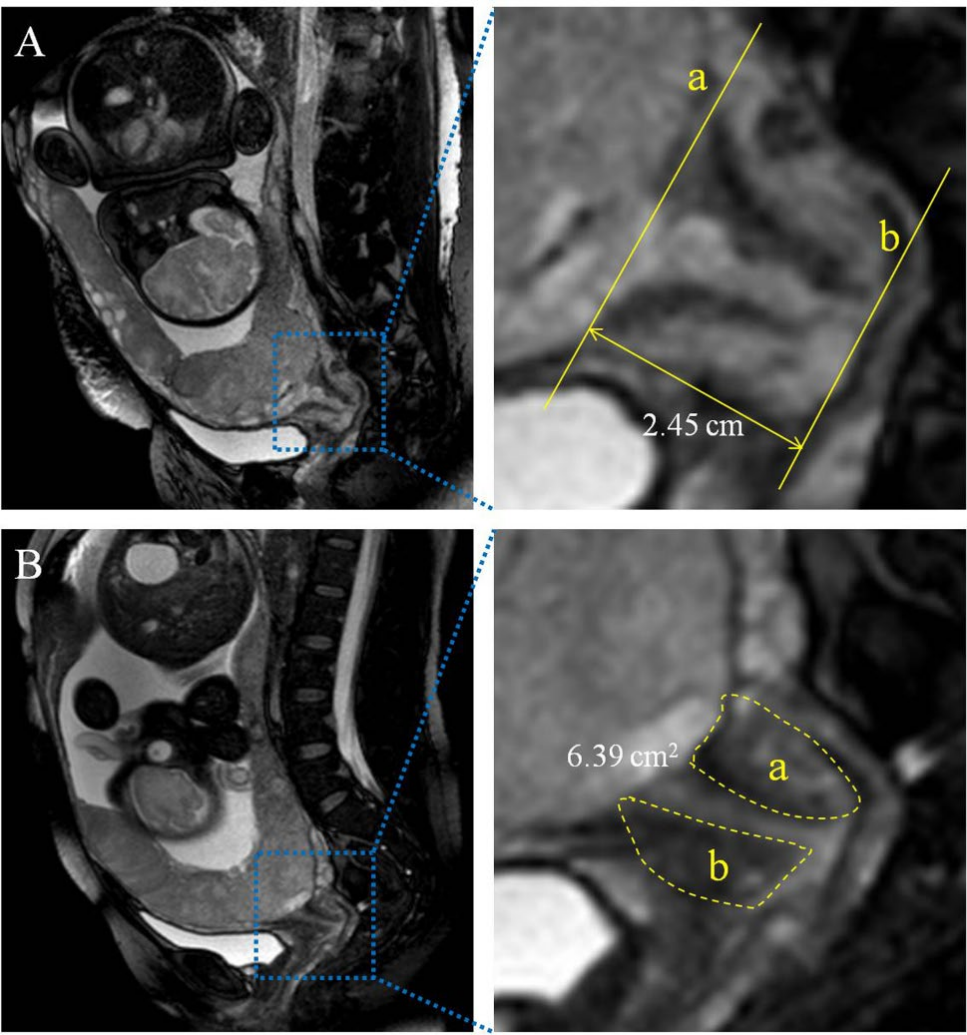
**A** Cervical length was measured between lines a and b in sagittal magnetic resonance imaging (MRI) image (2.45 cm). **B** Cervical area was measured by Image J software in sagittal MRI image (yellow circles a and b) (6.39 cm^2^)

The cervical length and cervical area were independently measured by 2 experienced radiologists (both with more than 10 years’ experience in obstetric imaging reading). The MRI images of PAS patients were imported into Image J software version 1.50 (National Institute of Health, Bethesda, USA) in order to measure cervical area and cervical length. The main steps were as follows: (1) Lines a and b are perpendicular to the cervical canal through the internal and external of cervical canal respectively, and the shortest distance between the two lines is cervical length. (2) The anterior and posterior lips of the cervix were traced by yellow dashed lines in the median sagittal position of MRI, and the cervical area (a + b) was calculated by Image J software (Fig. 1).

### Statistical analysis

The sample size was determined with independent samples t tests using PASS software (version 11.0.7 Windows, NCSS, LLC., Kaysville, UT, USA), a total of 158 samples would have 90% power to detect a difference between the two groups with a significance level of 0.05. The Kolmogorov-Smirnov normality test was used to determine whether continuous variables were normally distributed. Values were expressed as the mean ± standard deviation (SD) or median and interquartile range. Student’s t-test was used for normally distributed variables. Non-normally distributed numerical data were compared by Mann-Whitney test between the two groups. Categorical variables were presented with counts and proportions and their differences were compared by Chi-squared test. Multivariable logistic regression analysis was performed to identify MRI features that may contribute to MH risk. The correlation analysis between cervical length, cervical area and amount of blood loss was performed with Pearson analysis. Sensitivity, specificity, positive predictive value (PPV), and negative predictive value (NPV) were calculated using standard definitions. Receiver operating characteristic curve (ROC) was used to determine the cut-off of MRI signs with the best sensitivity and specificity for distinguishing MH in PAS patients.

Interobserver agreement and Kappa coefficients for interobserver reliability were calculated for each MRI sign according to the following definitions: 1.0 perfect agreement, 0.91 to 0.99 almost perfect agreement, 0.81 to 0.90 substantial agreement, 0.71 to 0.80 moderate agreement, 0.61 to 0.70 fair agreement, and ≤ 0.6 slight agreement. Statistical significance was defined as a *P* < 0.05 and all statistical analyses were performed with IBM SPSS statistics software (version 23.0, SPSS Inc., Chicago, IL, USA).

## Results

Out of 158 placenta previa patients with PAS, 73 had postpartum hemorrhage greater than 2000 ml and 85 had postpartum hemorrhage less than 2000 ml. General clinical information, including maternal age, BMI, gravidity, parity, number of abortions, number of previous cesarean delivery, previous history of placental previa, gestational age at MRI, prenatal vaginal bleeding, and hysterectomy showed no differences between the two groups. Gestational age at delivery and neonatal birth weight were significantly lower among pregnant women with MH than those without MH. The operation time, intraoperative blood loss and blood transfusion were significantly higher in Patients with MH. In terms of the types of PAS, patients with MH had more percreta types than patients without MH (*P* = 0.001) (Table 1).

**Table 1:** Patients clinical features of the study groups

Characteristic	Patients with MH (*n* = 73)	Patients without MH (*n* = 85)	Statistic	P value
Maternal age	32.206 ± 4.116	31.082 ± 4.195	1.692	0.093^a^
BMI (kg/m^2^)	26.016 ± 3.155	25.792 ± 4.115	0.379	0.705 ^a^
Gravidity	3(2,4)	3(2,4)	1.464	0.143^b^
Parity	2(2,2)	2(1,2)	1.541	0.123^b^
Number of abortions	1(1,2)	1(1,2)	0.819	0.413^b^
Number of previous cesarean delivery	1(1,2)	1(1,2)	1.706	0.088^b^
Previous history of placental previa	7 (9.589)	4 (4.706)	1.446	0.229^c^
Gestational age at MRI (weeks)	33.264 ± 2.150	33.784 ± 1.764	1.671	0.097^a^
Prenatal vaginal bleeding	25(34.247)	21(24.706)	1.732	0.188^c^
Gestational age at delivery (weeks)	35.240 ± 1.534	36.105 ± 1.043	4.192	0.001^a^
Neonatal birth weight (g)	2426.164 ± 376.536	2704.706 ± 449.446	4.182	0.001^a^
Operation time (min)	90.945 ± 28.915	78.464 ± 30.867	2.609	0.010^a^
Intraoperative blood loss (mL)	2480.548 ± 601.486	1259.024 ± 373.163	15.562	0.001^a^
Blood transfusion (mL)	1884.795 ± 603.453	792.471 ± 424.713	13.293	0.001^a^
ICU admission	15 (20.548)	6 (7.059)	6.200	0.013^c^
Hysterectomy	5 (6.849)	1 (1.176)	3.459	0.063^c^
The type of PAS			12.820	0.002^c^
Accreta	31 (42.466)	52 (61.176)		
Increta	25 (34.247)	29 (34.118)		
Percreta	17 (23.288)	4 (4.706)		

Note BMI, body mass index; MRI, magnetic resonance imaging; MH, massive hemorrhage; ICU, intensive care unit; PAS, placenta accreta spectrum

^a^Student’s t-test was performed

^b^Mann-Whitney test was performed

^c^Chi-squared test was performed

**Fig. 2:**
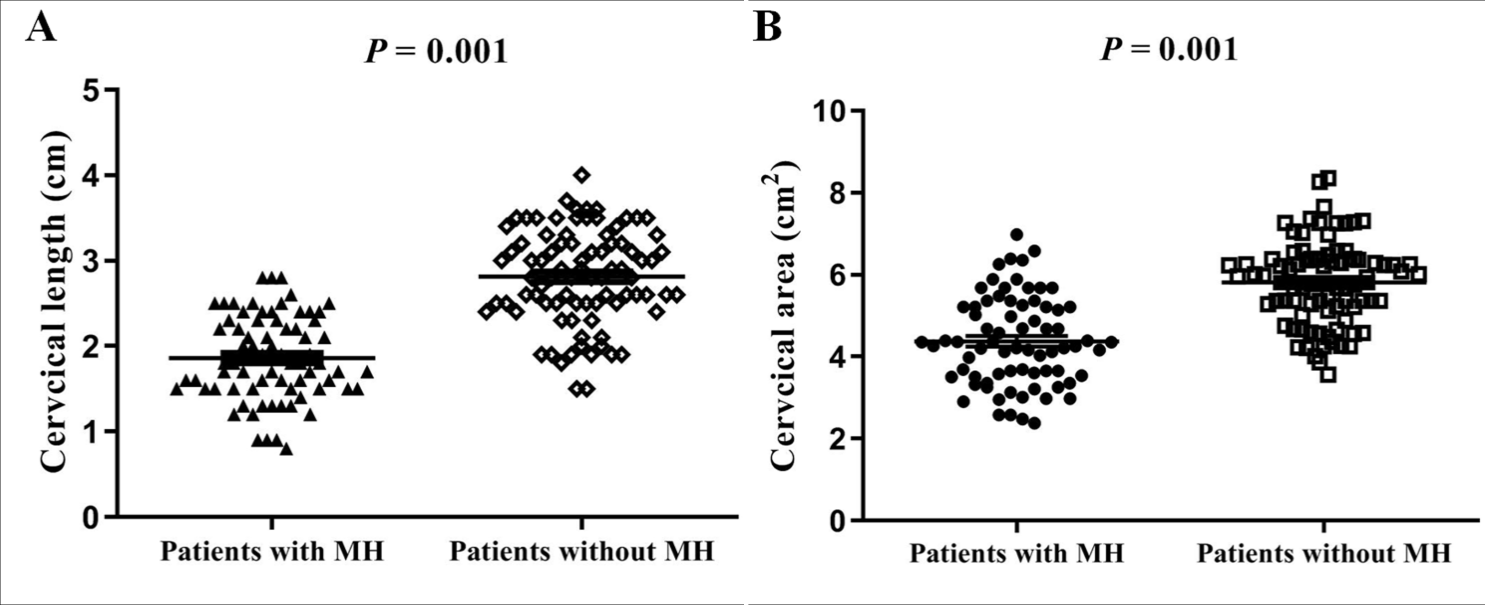
**A** Cervical length in patients with and without massive hemorrhage (MH). **B** Cervical area in patients with and without MH

**Fig. 3:**
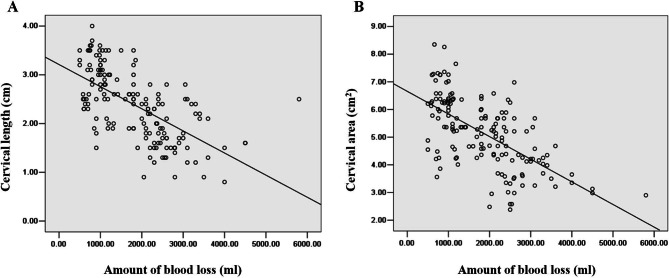
**A** Association between cervical length and amount of blood loss in placenta accreta spectrum disorders (PAS) patients (r=−0.613). **B** Association between cervical area and amount of blood loss in PAS patients (r=−0.629)

The interobserver variability for MRI findings was almost perfect agreement, and the kappa of cervical length and area were both greater than 0.900 (Table 2). The cervical length and area in patients with MH were both significantly smaller than those in patients without MH (Fig. 2). The results of multivariate analysis show that cervical length (OR, 3.406; 95% CI, 1.927–6.409; *P* = 0.001) and cervical area (OR, 5.374; 95% CI, 2.805–8.336; *P* = 0.001) were significantly associated with MH (Table 3). In all placenta previa patients with PAS, a negative linear was found between cervical length and amount of blood loss (r =−0.613), and between cervical area and amount of blood loss (r =−0.629), as demonstrated in Fig. 3.

**Table 2:** Interobserver reliability of magnetic resonance imaging (MRI) in the measurement of MRI features

MRI features	Either	All	Agree	Kappa	Interpertation
Cervical length	86 (54.430)	80 (50.633)	96.203	0.924	Almost perfect
Cervical area	82 (51.899)	75 (47.468)	95.570	0.911	Almost perfect

Kappa coefficients for interobserver reliability were performed

**Table 3:** Multivariate logistic regression analysis of risk factors for patients with MH

Variable	Multivariate analysis	
	OR (95%CI)	P^a^
Cervical length (cm)		0.001
> 3.00	1	
< 3.00	3.406 (1.927–6.409)	
Cervical area (cm^2^)		0.001
< 4.00	1	
> 4.00	5.374 (2.805–8.336)	

^a^Multivariable logistic regression analysis was performed

**Fig. 4:**
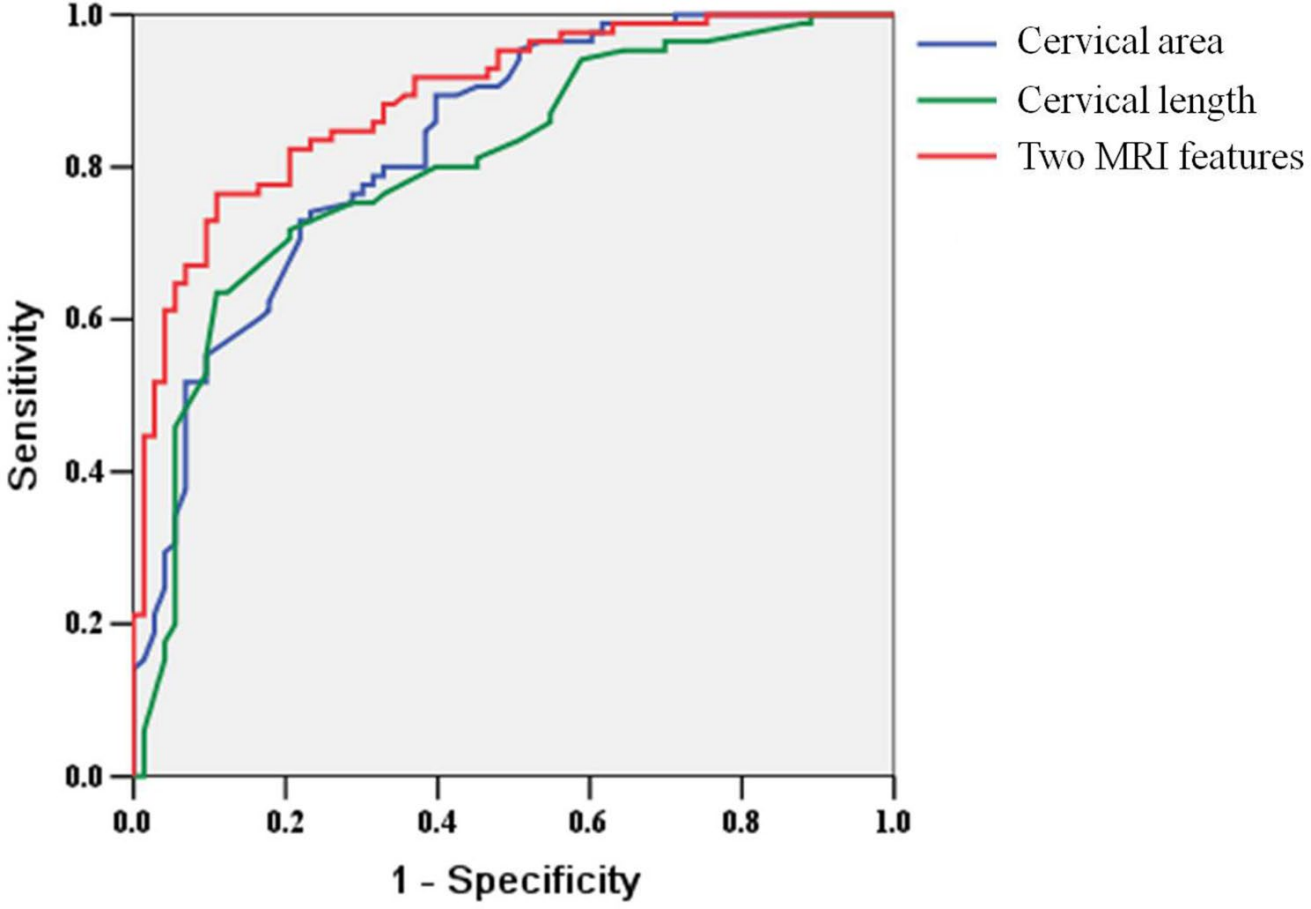
Receiver operating characteristic (ROC) curve of different magnetic resonance imaging (MRI) features in patients with placenta accreta spectrum disorders (PAS)

The results of sensitivity, specificity, PPV, NPV and area under the curve (AUC) value of each MRI sign regarding MH were shown in Table 4; Fig. 4. AUC of cervical length for the predictive MH was 0.804 with a cutoff of 30 mm (83.605% sensitivity and 86.328% specificity, *P* = 0.001). AUC of cervical area for the predictive MH was 0.832 with a cutoff of 4.0 cm^2^ (85.035% sensitivity and 84.254% specificity, *P* = 0.001). When combined with cervical length and cervical area, the sensitivity, specificity, and AUC for the predictive MH were 88.618%, 90.209%, and 0.890, respectively.

**Table 4:** Receiver operating characteristic analyses for prediction of massive hemorrhage based on cervical length and cervical area

Variable	Cut-off	Sensitivity % (95% CI)	Specificity % (95% CI)	PPV %	NVP %	P^a^
Cervical length (mm)	28	80.551 (73.895–86.806)	88.253 (81.952–94.239)	85.484	84.086	0.001
Cervical length (mm)	30	83.605 (77.283–88.626)	86.328 (80.247–92.038)	84.297	86.018	0.001
Cervical length (mm)	32	85.450 (78.028–89.509)	83.159 (78.382–90.426)	81.335	86.936	0.001
Cervical area (cm^2^)	3.5	83.214 (78.024–89.482)	87.322 (81.206–92.519)	84.933	85.830	0.001
Cervical area (cm^2^)	4.0	85.035 (79.254–88.329)	84.254 (78.291–89.038)	82.263	86.765	0.001
Cervical area (cm^2^)	4.5	86.958 (81.219–91.560)	82.359 (77.084–86.690)	80.892	88.028	0.001

Note PPV, positive predictive value; NPV, negative predictive value

^a^Receiver operating characteristic curve (ROC) was performed

## Discussion

Maternal hemorrhage is an important cause of maternal death, especially in developing countries [14]. Multiple factors can lead to MH, and placenta previa with PAS is one of the worst obstetric complication that is associated with severe, life-threatening maternal hemorrhage. In this study, we described a method of identifying MH in placenta previa patients with PAS by measuring the cervical length and cervical area. The cervical length and cervical area are objective indicators that can be quantified, and artificial intelligence could be used to measure and analyze the risk of MH in placenta previa patients with PAS during caesarean section in the future. Prospective study had shown that placenta previa patients with short cervix could predict a higher risk of severe hemorrhage during cesarean Sect. [15]. The exact mechanism is not clear, and some reports speculate that it may be related to inelastic cervix in the absence of muscle tissue and high vascularity over smaller surface area of short cervix [10, 16]. The cutoff of cervical length used to predict adverse perinatal outcomes varies from literature to literature. In this study, 30 mm as the cutoff value for cervical length was appropriate because it had high sensitivity and specificity to identify MH, which was consistent with the study reported by Zaitoun et al [9].

Some previous studies have reported that ervical length combined with other MRI signs was superior than a single measurement in predicting adverse perinatal outcomes [9, 17]. There were no relevant studies on the cervical area of placenta previa in previous studies. In this study, it was found that the cervical area of MH group was significantly smaller than that of control group, and the mechanism is still unclear. Our results demonstrate that, in placenta previa patients with PAS, cervical area less than 4.00 cm^2^ was associated with an increase in the number of operation time, intraoperative blood loss, and blood transfusion. Placenta previa patients in the MH group had lower neonatal weight, which might be related to earlier gestational weeks of delivery. Some studies have revealed similar results [18, 19].

Therefore, this study suggested that shorter cervical length and smaller cervical area would adversely affect the clinical outcomes in operations in placenta previa patients with PAS. The intact cervix can be seen in the MRI sagittal plane without PAS, and if the placenta is implanted in the cervix, the cervix will be irregular in shape and smaller in size. In combination with our study results, we believe that the placenta tissue in the lower part of the uterus is hyperplasia and enlargement and pulls the cervix to shorten the cervix, and the placenta tissue of some patients intruded into the cervical canal to thin the cervical tissue and eventually lead to the smaller cervical area in placenta previa patients with PAS. Vital organs around the cervix and progressive narrowing of the operative field are the major disadvantages of the shorter cervical length and smaller cervical area. There are vital organs around the cervix such as bladder, ureter and rectum, uterine bleeding and implanted placental tissue will obscure the operative field, and the operative field is narrow, all of which are disadvantages for operation in patients with shorter cervical length and smaller cervical area [10]. In this stdudy we found that the optimal cutoff value to best identify MH during caesarean section were cervical length < 3.00 cm and cervical area > 4.00 cm^2^. Placenta previa patients had a shorter cervical length and smaller cervical area, which usually meaned that the placenta was implanted in the cervical canal with vascular hyperplasia. Karaşin SS investigated the effects of cervix thickness on labor, and the cervical length/thickness ratio was a good predictor of cervical ripening activity. Therefore, the relationship between cervix thickness and MH might be wroth studying in placenta previa patients with PAS in future studies [20]. MRI had high accuracy in identifying MH, it may help obstetricians choose the appropriate therapeutic schedule and improve patient prognosis.

### Limitations

This study has several limitations. First of all, this study was a small sample study, and the results obtained by a large sample study with multiple centers were more accurate. Secondly, due to retrospective analysis, some bias might inevitably affect the results of statistical analysis. Finally, these pregnant women included in this study were screened by ultrasound for a high risk of MH and then had an MRI, thus, was not representative of the general obstetric population.

## Conclusion

In summary, the performance of cervical length and cervical area by antenatal MRI was associated with MH and total blood product transfusion. The cervical length and cervical area might be used as good indicators to predict the risk of MH in placenta previa patients with PAS during caesarean section.

## Data Availability

All data generated or analysed during this study are included in this published article.
